# Quantum tunnelling of the magnetisation in single-molecule magnet isotopologue dimers†
†Electronic supplementary information (ESI) available. CCDC 1898383 and 1898384. For ESI and crystallographic data in CIF or other electronic format see DOI: 10.1039/c9sc01062a


**DOI:** 10.1039/c9sc01062a

**Published:** 2019-04-15

**Authors:** Eufemio Moreno-Pineda, Gheorghe Taran, Wolfgang Wernsdorfer, Mario Ruben

**Affiliations:** a Institute of Nanotechnology (INT) , Karlsruhe Institute of Technology (KIT) , Hermann-von-Helmholtz-Platz 1 , D-76344 Eggenstein-Leopoldshafen , Germany . Email: eufemio.pineda@kit.edu ; Email: wolfgang.wernsdorfer@kit.edu ; Email: mario.ruben@kit.edu; b Physikalisches Institut , Karlsruhe Institute of Technology , D-76131 Karlsruhe , Germany; c CNRS , Institut Néel , F-38042 Grenoble , France; d Institut de Physique et Chimie des Matériaux de Strasbourg (IPCMS) , CNRS-Université de Strasbourg , 23 rue du Loess, BP 43 , F-67034 Strasbourg Cedex 2 , France

## Abstract

Quantum tunnelling of the magnetisation plays a major role in the magnetic properties of lanthanide Single-Molecule Magnets: while it is considered a problem for data storage device applications since it leads to information loss, it is an essential pre-requisite for the read-out and manipulation of the nuclear states in Quantum Information Processing schemes. Here we describe two isotopologue dysprosium dimers, *i.e.* [(^163^Dy(tmhd)_3_)_2_(bpym)] and [(^164^Dy(tmhd)_3_)_2_(bpym)] (tmd = tris(tetramethylheptanedionato) and bpym = bipyrimidine), where the nuclear spin presence or absence clearly affects the magnetic properties of the systems. Through μ-SQUID studies at milli-Kelvin temperatures and alternating current magnetic measurements, we find significant differences in the magnetic behaviour of both complexes. While simulation of the hysteresis loops at 30 mK reveals that the presence of nuclear spin does not influence the tunnelling rate, we find that it facilitates the coupling to the phonon bath enhancing the direct relaxation process; an observation reflected in the temperature and field dependence of the relaxation rates.

## Abstract 2

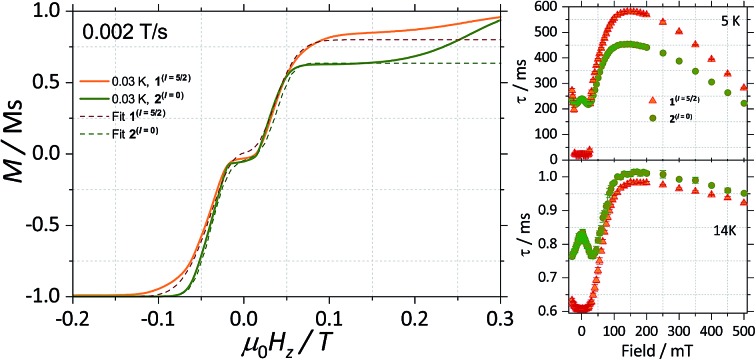
Low-temperature magnetic studies of two isotopologues dimers, with and without nuclear spins, reveal that, at very low temperatures, the nuclear spin facilitates the coupling to the phonon bath enhancing the direct relaxation process; observation reflected in the temperature and field dependence of the relaxation rates, whilst at higher temperatures the effect of the nuclear spins is less relevant.

## Introduction

The highly anisotropic character of lanthanides and the strong effect of the ligands chelating the lanthanide ions resulted in the observation of mononuclear molecules exhibiting slow relaxation of the magnetisation, namely Single-Ion Molecule Magnets (SIMs), a subclass of Single-Molecule Magnets (SMMs).^1^,^2^ The strong anisotropy and large energy barriers, along with quantum effects, have led to their proposed use in several technological applications ranging from data storage devices to quantum bits for quantum computers.^3^ Depending on the desired application different characteristics are required. For example, large energy barriers to the reversal of the magnetisation (*U*_eff_) and diminished Quantum Tunnelling of the Magnetisation (QTM) rates are necessary for data storage devices, whilst for the implementation of lanthanide-containing SMMs (Ln-SMMs) as qubits, an isolated electronic ground doublet state along with QTM provides access to the nuclear spins embedded in the lanthanide metal ion.3a,^4^

On the data storage device side, a large separation between the ground state and the first excited state would allow the molecule to preserve the stored information at high temperatures. In this regard, scientists have gained a deep insight into the general pre-requisites necessary for the design of molecules possessing large energy barriers; clearly exemplified by several molecules possessing extremely large *U*_eff_.^5^ Unfortunately, despite the large *U*_eff_ the magnetic properties of SMMs are often hampered by the QTM, effect that allows the electronic spins to tunnel through the energy barrier following a non-thermally activated pathway. In turn, although large *U*_eff_ can be obtained, in most cases the hysteresis loops of Ln-SMMs are practically closed at zero field.^6^ As consequence, out of the many Ln-SMM reported up today, molecules exhibiting large magnetic hysteresis remain scarce.^7^‡
‡Crystallographic data for **1**^(*I* = 5/2)^ [C_78_H_130_Dy_2_N_4_O_14_]: Mr = 1672.85, triclinic, *T* = 180.0(2) K, *a* = 10.8743(2), *b* = 13.8790(3), *c* = 14.8618 (4) Å, *α* = 90.039(2)°, *β* = 93.333(2)°, *γ* = 107.553(2)°, *V* = 2131.63(9) Å^3^, *Z* = 1, *ρ* = 1.303 g cm^–3^, total data = 25289, independent reflections 8697 (Rint = 0.0289), *μ* = 1.797 mm^–1^, 467 parameters, *R*_1_ = 0.0256 for *I* ≥ 2*σ*(*I*) and w*R*_2_ = 0.0630. Crystal data for **2**^(*I* = 0)^ [C_78_H_130_Dy_2_N_4_O_13_]: Mr = 1672.85, triclinic, *T* = 180.0(2) K, *a* = 10.8671(2), *b* = 13.8736(3), *c* = 14.8582(3) Å, *α* = 91.995(2)°, *β* = 93.347(2)°, *γ* = 107.530(2)°, *V* = 2129.20(8) Å^3^, *Z* = 1, *ρ* = 1.305 g cm^–3^, total data = 24512, independent reflections 8663 (Rint = 0.0325), *μ* = 1.797 mm^–1^, 480 parameters, *R*1 = 0.0241 for *I* ≥ 2*σ*(*I*) and w*R*_2_ = 0.0610. Single crystal X-ray diffraction data of **1**^(*I* = 5/2)^ and **2**^(*I* = 0)^ was collected employing a STOE StadiVari 25 diffractometer with a Pilatus300 K detector using GeniX 3D HF micro focus with MoKα radiation (*λ* = 0.71073 Å). The structure was solved using direct methods and was refined by full-matrix least-squares methods on all F2 using SHELX-2014^25^ implemented in Olex2.^26^ The crystals were mounted on a glass tip using crystallographic oil and placed in a cryostream. Data were collected using φ and ω scans chosen to give a complete asymmetric unit. All non-hydrogen atoms were refined anisotropically. Hydrogen atoms were calculated geometrically riding on their parent atoms. Full crystallographic details can be found in CIF format: see the Cambridge Crystallographic Data Centre database (CCDC 1898383 and 1898384 for **1**^(*I* = 5/2)^ and **2**^(*I* = 0)^, respectively).


In spite the harmful effects for data storage device applications, QTM has been shown to play an important role in the successful implementation of SMMs in quantum information processing (QIP) schemes, where the nuclear spins embodied in the lanthanide are utilised as quantum registers.^4^ In the nuclear spins scheme, the highly anisotropic character of the SMM isolates the ground doublet state, which is thenceforth coupled to the nuclear spins embedded in the lanthanide by the strong hyperfine interaction; thus, the ground doublet state splits into (2*I* + 1) states, where *I* is the nuclear spin of the lanthanide. At some of these crossings QTM is active, consequently allowing the read-out and manipulation of the states of the qubit.^4^ Remarkably, the multilevel character of the nuclear states contained in the lanthanides allows the operation of several states in a single unit. Systems possessing these characteristics are termed “qu*d*its”, where *d* represent the number of active states.^8^

In both schemes, it is clear that QTM, as well as spin–lattice interactions, plays a major role on the magnetic behaviour of the molecular systems,^9^ hence for the successful implementation of Ln-SMMs in any of these two applications, a deep understanding of relaxation effects is required. This has been evidenced by studies employing isotopically enriched lanthanide sources in mononuclear SMMs with moderate to high energy barriers, where nuclear spins are not entirely responsible for the observed fast tunnelling rates.^10^,^11^

Herein, we study the effect of the nuclear spins on the dynamic properties of two isotopically enriched dysprosium dinuclear SMMs *via* AC magnetic susceptibility studies as well as single crystal μ-SQUID data at sub-Kelvin temperatures. We find the tunnelling probability to be equal for both isotopologue compounds; therefore, the effect of the nuclear spins is to span the avoided crossings over a larger field range. Our results agree with recent reports of QTM studies of high energy barriers SMMs.^11^ Nonetheless, although nuclear spins do not affect the QTM rate, we find that these enhance the spin–phonon coupling, increasing the direct relaxation process in the SMMs.

## Results and discussions

### Syntheses and structures

**Fig. 1:**
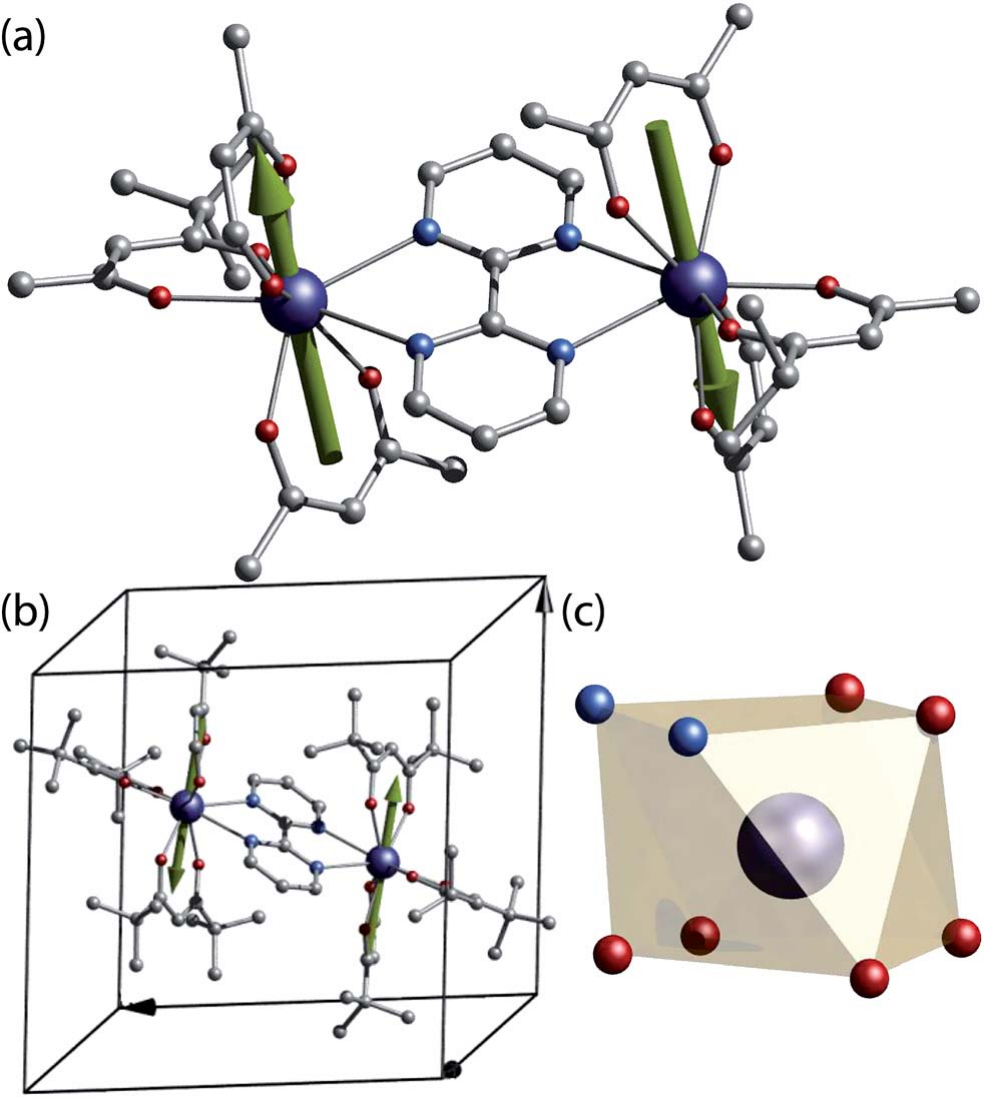
(a) Crystal structure of [(Dy(tmhd)_3_)_2_(bpym)]. (b) Unit cell of the [(Dy(tmhd)_3_)_2_(bpym)] complex showing a single molecule residing in the unit cell. (c) Polyhedral representation of the Dy(iii) site on a N_2_O_4_ geometry. Colour code: C, grey; N, cyan; O, red; Dy, dark blue. Green arrows represent the anisotropy axis for each Dy(iii) obtained from *ab initio* CASSCF calculations.

For our study a known SMM dinuclear complex was chosen.^12^ The complexes can be obtained by reacting one equivalent of bipyrimidine (bpm) ligand with two equivalents of the respective Ln(tmhd)_3_(H_2_O)_2_ precursor (where Ln = ^163^Dy^3+^ (*I* = 5/2), ^164^Dy^3+^ (*I* = 0) and tmhd = tris(tetramethylheptanedionato)) in absolute ethanol. X-ray quality crystals are grown from a dichloromethane/ethanol solution. The isotopologue complexes feature two neutral dinuclear systems with formula [(^163^Dy(tmhd)_3_)_2_(bpym)] (**1**^(*I* = 5/2)^) and [(^164^Dy(tmhd)_3_)_2_(bpym)] (**2**^(*I* = 0)^) (Fig. 1a), as revealed from X-ray single crystal studies.‡

Both isostructural complexes **1**^(*I* = 5/2)^ and **2**^(*I* = 0)^ crystallise in the triclinic *P*1[combining macron] space group, with half-molecule in the asymmetric unit, thus, both dysprosium ions are related by an inversion centre. A single molecule resides in the unit cell (Fig. 1b). These characteristics are of importance for the understanding of the single crystal μ-SQUID data (*vide infra*).

At the metal side, each metal ion possesses a N_2_O_4_ coordination geometry formed by six oxygen atoms from the tmhd and two nitrogen atoms of the bpm (Fig. 1c). The Dy···O distances range between 2.263(2) Å to 2.335(2) Å for **1**^(*I* = 5/2)^ and 2.264(3)–2.334(1) Å for **2**^(*I* = 0)^, whilst the Dy···N distances in both cases are longer, with values ranging from 2.586(2) Å to 2.609(2) Å for **1**^(*I* = 5/2)^ and 2.587(2) Å to 2.609(2) Å for **2**^(*I* = 0)^. The intramolecular Dy···Dy distance are 6.7964(4) Å and 6.7971(4) Å for **1**^(*I* = 5/2)^ and **2**^(*I* = 0)^, respectively. The coordination geometry around the dysprosium ions can be best described as a square antiprism with a continuous shape measure (CShM) of 0.607 for **1**^(*I* = 5/2)^ and 0.615 for **2**^(*I* = 0)^ (see ESI, Table S2†).^13^

### *Ab initio* CASSCF-SO calculations

To develop a detailed picture of the electronic structure of **1**^(*I* = 5/2)^ and **2**^(*I* = 0)^ and to rationalise their magnetic properties, Complete Active Space Self-Consistent Field spin–orbit calculations of the CASSCF/SO-RASSI/SINGLE_ANISO^16^–^19^ type were performed (see ESI† for details). Prediction of the electronic structure of the individual Dy(iii) ions yields an isolated doublet ground state characterised by highly axial *g* tensors, *i.e. g*_xx_ = *g*_yy_ ≈ 0 and *g*_zz_ ≈ 20. The low-lying ligand field states have the following order: *m*_J_ = ±15/2, ±13/2, ±11/2, ±9/2, with relative energies of 0, 188, 270, 310 K, respectively. The ensuing excited states are highly mixed and bunched over 380 to 730 K. Due to the site symmetry of the Dy(iii) ions in **1**^(*I* = 5/2)^ and **2**^(*I* = 0)^, the anisotropic magnetic axes are parallel (see Fig. 1a). In addition, the average values of the matrix elements of magnetic moment connecting the electronic states (Fig. S4†) show lower tunnelling rates between the ground doublet |±15/2) show lower tunnelling rates between the ground doublet |±15/2〉 state, while higher transition rates occur states at higher energy. In turn, the most probable thermally activated relaxation pathway would involve spin–phonon excitation to the first, second and third excited doublets, followed by relaxation to the opposing ground state. The highly axial character of the ground state obtained by CASSCF agrees with the observed SMM behaviour for the non-isotopically enriched analogue and complexes here studied ( state, while higher transition rates occur states at higher energy. In turn, the most probable thermally activated relaxation pathway would involve spin–phonon excitation to the first, second and third excited doublets, followed by relaxation to the opposing ground state. The highly axial character of the ground state obtained by CASSCF agrees with the observed SMM behaviour for the non-isotopically enriched analogue and complexes here studied (*vide infra*).^12^

### Low-temperature μ-SQUID studies

**Fig. 2:**
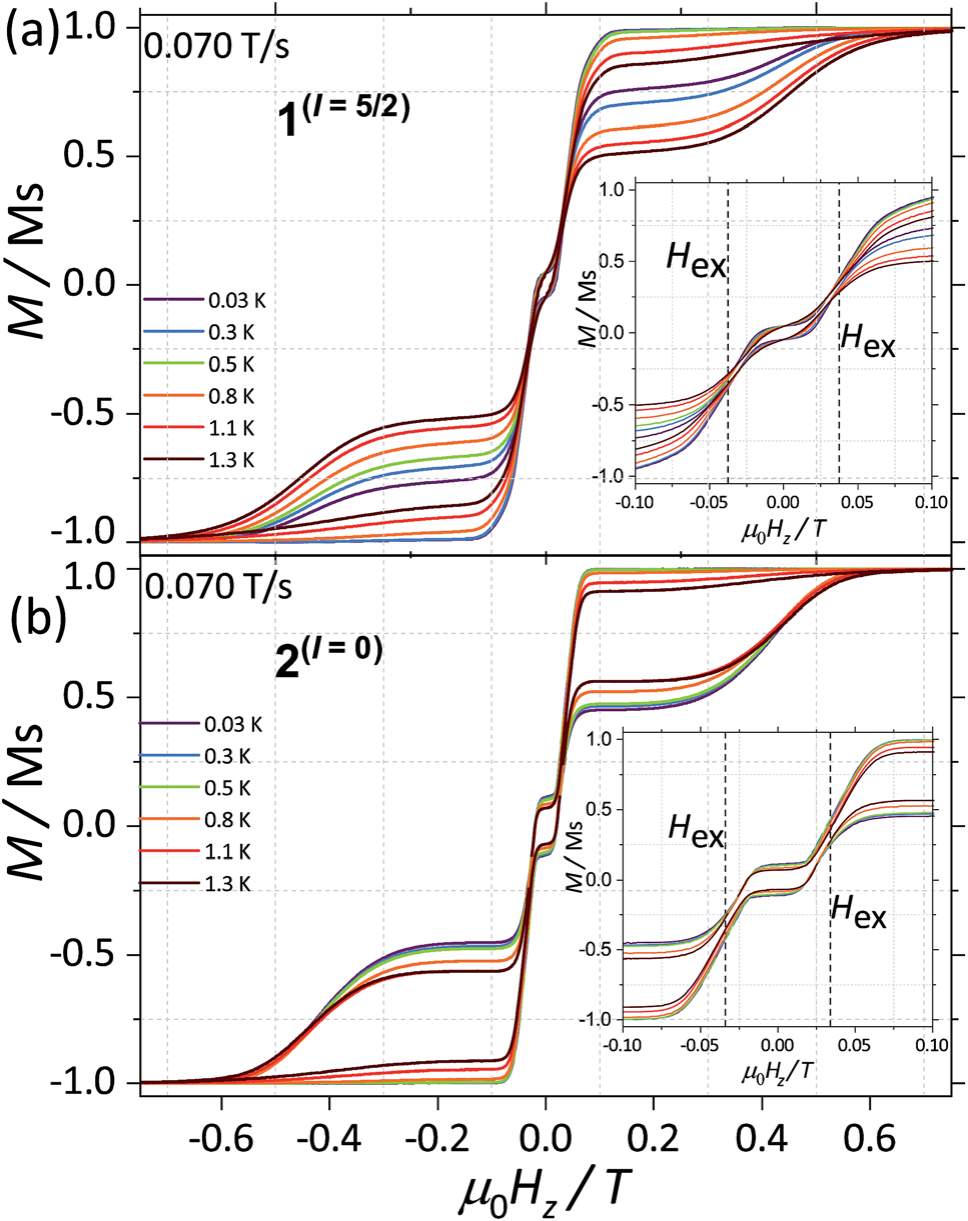
Temperature dependence of the magnetisation of (a) **1**^(*I* = 5/2)^ and (b) **2**^(*I* = 0)^ at a field sweep rate of 0.070 T s^–1^. The field was applied parallel to the easy axis of the magnetisation. Before each field sweep, a waiting time of more than 1000 s at ±1 T was used to thermally equilibrate the nuclear spin system with the thermal bath.

CASSCF calculations predict **1**^(*I* = 5/2)^ and **2**^(*I* = 0)^ to be SMMs, with relaxation pathways active through the first, second and third excited states. In order to understand the relaxation dynamics of the complexes, and to minimise the complexity of the possible relaxation pathways taking place in the SMMs, we first investigate the nuclear spin effect on the magnetic properties of **1**^(*I* = 5/2)^ and **2**^(*I* = 0)^*via* μ-SQUID studies at very low temperatures, where thermally activated processes are expectedly less effective. μ-SQUID measurements were performed on single crystals of **1**^(*I* = 5/2)^ and **2**^(*I* = 0)^ with the field applied along the main anisotropic axis, employing the transverse method.^14^ Hysteresis loops studies were performed at different sweep rates and temperatures (Fig. 2 and S4†). Well-resolved two-steps hysteresis loops were obtained for **1**^(*I* = 5/2)^ and **2**^(*I* = 0)^ with the width of the hysteresis loops increasing with decreasing temperatures and increasing sweep rates, confirming the SMM behaviour of the complexes.

The loops are very typical for two antiferromagnetically coupled Ising-like spins: around zero field, the loops have a S-shape with two sharp tunnel steps at positive and negative fields. Above *μ*_0_*H*_Z_ = ±0.3 T, the loops have a broad step, which is strongly field-sweep-rate dependent and is a consequence of the direct relaxation process between the antiferromagnetic and ferromagnetic spin states. Additionally, the loops exhibit a small hysteresis at *μ*_0_*H*_Z_ = 0, which comes from the fact that some of the molecules do not tunnel to the antiferromagnetic ground state but remain pinned to the ferromagnetic state.^15^ Upon simple comparison of the hysteresis curves for **1**^(*I* = 5/2)^ and **2**^(*I* = 0)^ it can be observed that narrower loops are obtained for the nuclear spin bearing system, indicating the relaxation mechanism is more effective for this system. Note also that the loops for **1**^(*I* = 5/2)^ show a more temperature dependent behaviour than that of **2**^(*I* = 0)^.

The mean exchange field (*H*_ex_) can be directly extracted from the inflexion points in the hysteresis loops, leading to an effective exchange constant between the Ising spins of the Dy(iii) ions: *H*_ex_ = *J m*_J_/*g*_J_*μ*_B_ where *m*_J_ = 15/2 and *g*_J_ = 4/3. The determined *H*_ex_ (4.18 mK) is slightly larger than the one obtained from a purely point dipolar approximation: *D*dipzz = 3.53 mK for a ^163^Dy···^163^Dy distance of 6.7964(4) Å and ^164^Dy···^164^Dy distance of 6.7971(4) Å, thus the interaction between the Dy(iii) pairs is mainly of dipolar origin, with a small exchange contribution. Note that the shortest Dy···Dy distance is 9.9374(5) Å and 9.9306 (5) Å for **1**^(*I* = 5/2)^ and **2**^(*I* = 0)^, respectively, therefore, intermolecular interactions are less relevant compared to intramolecular.

**Fig. 3:**
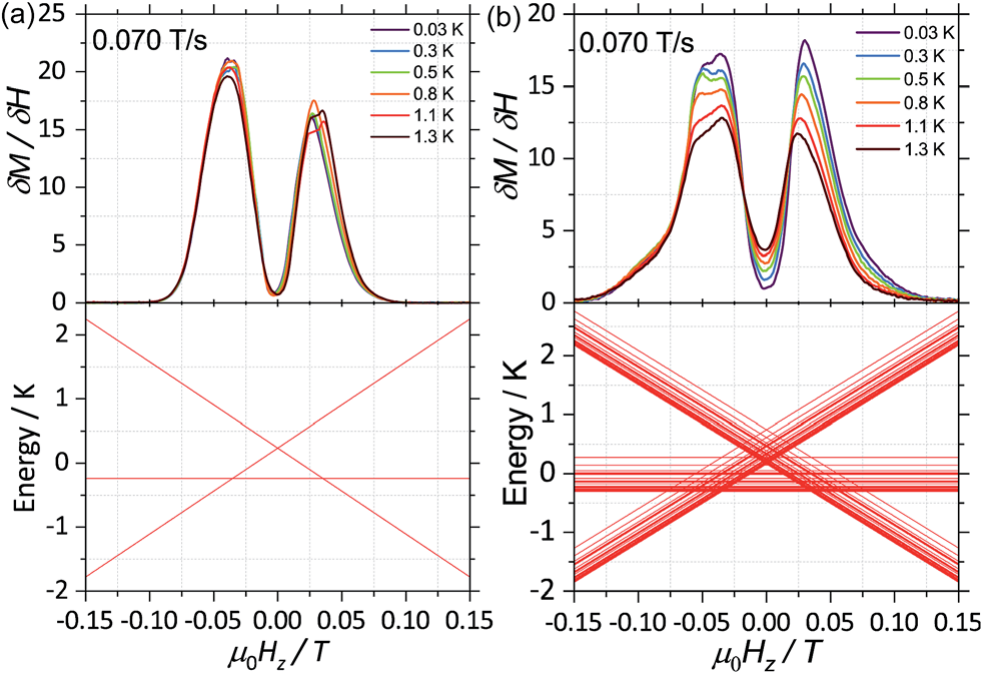
First field derivative for a field sweep from –1 T to +1 T of the data in Fig. 2 for (a) **2**^(*I* = 0)^ and (b) **1**^(*I* = 5/2)^. Bottom panels in (a) and (b) are the simulated Zeeman diagram with the field parallel to the easy axes, employing (1) (for **2**^(*I* = 0)^) and (2) (for **1**^(*I* = 5/2)^) and parameters described in the text.

With the knowledge of the low-lying magnetic properties of **1**^(*I* = 5/2)^ and **2**^(*I* = 0)^, it is possible to understand the precise role of the absence/presence of the nuclear spins in both complexes. To begin with our analysis, we firstly focus on the low-temperature magnetic properties of the individual **2**^(*I* = 0)^, as the lack of nuclear spins embodied in the ^164^Dy(iii) ions simplifies the analysis. The single ion magnetic properties of the Dy(iii) dimers are dominated by the spin–orbit coupling and the interaction with the surrounding ligands, leading to a separation of 188 K between the ground *m*_J_ = ±15/2 and the first excited, *m*_J_ = ±13/2, multiplet (see CASSCF section). This allows us to describe the complex as two isolated Ising spins (*s* = ½) coupled through an effective interaction *J*_eff_*σ*_1*z*_*σ*_2*z*_, where *J*_eff_ is an effective coupling that can incorporate a small exchange contribution and *σ*_1*z*,2*z*_ are the *z*-Pauli matrices. Thus, under the action of an external magnetic field applied along the easy axis, the Hamiltonian is written as:1
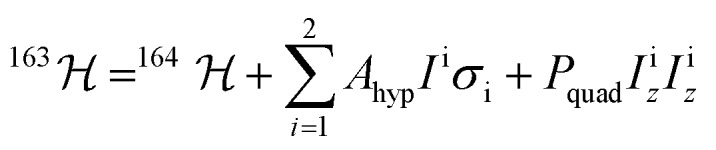
where *g*_eff_ = 20, and *Δ* is the effective tunnel splitting that arises from transverse interactions in the system. Fig. 3a shows the corresponding Zeeman diagram.

**Fig. 4:**
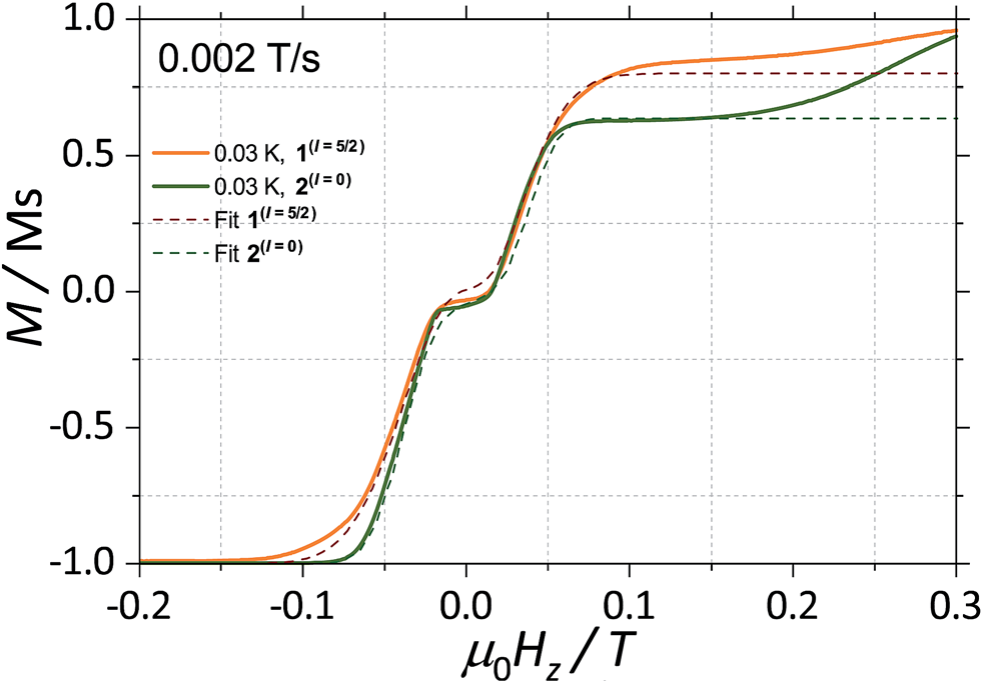
Fits of the magnetisation curves of **1**^(*I* = 5/2)^ (orange trace) employing eqn (2) and **2**^(*I* = 0)^ (green trace) employing eqn (1). The tunnelling probabilities were found to be *p* = 0.76 and 0.74, for **1**^(*I* = 5/2)^ and **2**^(*I* = 0)^, respectively.

With this, we can start to understand the hysteresis loops of the **2**^(*I* = 0)^ complex (Fig. 3a and 4). At *H*_z_ = –1 T (with *O*_*z*_ chosen along the easy axis of the Dy(iii) ions) the sample is polarised and all the spins are in the ground state |+15/2,+15/2) ions) the sample is polarised and all the spins are in the ground state |+15/2,+15/2〉. As the magnetic field is swept, the molecules remain in the ground state until the external field compensates the bias field, . As the magnetic field is swept, the molecules remain in the ground state until the external field compensates the bias field, *μ*_0_*H*_r_ ∼ –35 mT, and the SMM makes a transition from the ferromagnetic to the antiferromagnetic order by quantum tunnelling. The effective coupling was fixed to 4.18 mK, as described above. The height of the relaxations step (Δ*M*) is related to the tunnelling probability (*p*) through the relation: *p* = Δ*M*/(2*M*_in_), where *M*_in_ is the initial magnetisation. The next transition happens at *μ*_0_*H*_z_ ∼ +35 mT where the molecules relax non-adiabatically from the state |+15/2,–15/2 ∼ +35 mT where the molecules relax non-adiabatically from the state |+15/2,−15/2〉 to |−15/2,−15/2〉, with the same probability, to |–15/2,–15/2 ∼ +35 mT where the molecules relax non-adiabatically from the state |+15/2,−15/2〉 to |−15/2,−15/2〉, with the same probability, , with the same probability, *p*. The above discussion is valid only for the idealised situation describing a system of isolated molecules. In a real crystal the molecules are coupled by weak dipolar (and sometimes exchange) interactions and collective effects, such as reshuffling of the internal fields, which have an important influence on the relaxation process.10*f* Therefore, in order to properly describe the dynamics of the ensemble of SMMs, a multi-body model should be employed. However, in a first approximation, we can assume that the resonance fields of the molecules that tunnel follow a Gaussian distribution around the bias field, *μ*_0_*H*_r_, (Δ*N* ∼ exp(–(*μ*_0_*H*_z_ – *μ*_0_*H*_r_)2/*σ*^2^)), with the variance of this distribution depending linearly on the magnetisation of the sample: *σ*(*H*) = *σ*_0_|*M*(*H*)| + *σ*_min_. Using the above assumptions, we are able to fit the magnetisation curves, employing a nonlinear least-square algorithm (green trace in Fig. 4), with the sole fit parameter being the tunnelling probability, *p*, which for the sweeping rate of 2 mT s^–1^ is found to be *p* = 0.74. The parameters *σ*_0_ and *σ*_off_ that describe the distribution of the resonance fields are chosen so that a simultaneous fit of the magnetisation curves under different sweeping rates is obtained.

With a clear picture of the nuclear spin free system, now we are prepared to consider **1**^(*I* = 5/2)^. The ^163^Dy(iii) isotope has a nuclear magnetic moment *I* = 5/2 coupled to the electronic shell by the hyperfine (*A*_hyp_*Iσ*) and quadrupolar interaction (*P*_quad_*I*_*z*_^2^). Thus, the total Hamiltonian of the **1**^(*I* = 5/2)^ complex can be written as:2
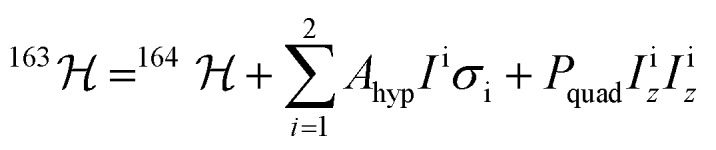
with *A*_hyp_ = 107.1 mK and *P*_quad_ = 19.6 mK. The corresponding Zeeman diagram is shown in Fig. 3b.

The analysis of the magnetisation curve of the **1**^(*I* = 5/2)^ complex is done in a similar fashion to the analysis of the **2**^(*I* = 0)^ complex, with two new assumptions related to the presence of the nuclear spin. We consider that the hyperfine levels corresponding to the ground multiplet |+15/2,+15/2 complex, with two new assumptions related to the presence of the nuclear spin. We consider that the hyperfine levels corresponding to the ground multiplet |+15/2,+15/2〉 are initially uniformly populated and the tunneling transitions are allowed only between the levels that conserve the nuclear spin, with a fixed probability are initially uniformly populated and the tunneling transitions are allowed only between the levels that conserve the nuclear spin, with a fixed probability *p*. The resulting fit is shown in Fig. 4 (orange trace), which yields the tunnelling probability, *p* = 0.76, for the sweeping rate of 2 mT s^–1^.

As a result, we observe that the magnitude of the tunnelling probability (*p*) for both compounds does not change (the small difference may originate in the difference in size and shape of the sample). At very low temperatures (*T* < 0.3 K), the nuclear spins have the sole role of broadening the relaxation steps. At higher temperatures, the hysteresis loops of the **1**^(*I* = 5/2)^ complex show a stronger temperature dependence than the **2**^(*I* = 0)^ ones (Fig. 2). This suggests that the spin lattice relaxation processes^20^–^22^ are greatly enhanced by the presence of the nuclear spin in the **1**^(*I* = 5/2)^ compound (*vide infra*).

### High-temperature static and dynamic magnetic studies

In order to get further insight into the role played by the nuclear spins in the relaxation process of the two isotopologues, we turn to direct current (DC) and alternating current (AC) susceptibility measurements. Static magnetic measurements were carried out employing restrained polycrystalline samples of **1**^(*I* = 5/2)^ and **2**^(*I* = 0)^ under an applied field of 1000 Oe, while the reported AC measurements are performed on polycrystalline samples and under an oscillating field of 3.5 Oe. The room temperature *χ*_M_*T* for the complexes shows similar values, *i.e.* 28.6 and 28.4 cm^3^ mol^–1^ K for **1**^(*I* = 5/2)^ and **2**^(*I* = 0)^, respectively. The values bode well with the expected ones for two isolated Dy(iii), *i.e.* 28.3 cm^3^ K mol^–1^ for two Dy(iii) with *J* = 15/2 and *g*_J_ = 4/3 (see Fig. S6†). Upon cooling, the *χ*_M_(*T*) profile for both complexes stays practically constant down to *ca.* 80 K when it starts decreasing. Below 5 K *χ*_M_(*T*) rapidly drops to a minimum value of 17.1 cm^3^ K mol^–1^ for **1**^(*I* = 5/2)^ and 15.4 cm^3^ K mol^–1^ for **2**^(*I* = 0)^, indicative of depopulation of crystal field levels and antiferromagnetic interactions. As can be observed in Fig. S5,† employing the *ab initio* results and the lines model, we are able to reproduce very well the *χ*_M_*T*(*T*) (see ESI† for details).

We investigate both the dynamic temperature dependence of the susceptibility under a constant frequency, *χ*(*T*;*ν*), and the frequency dependence under a fixed temperature, *χ*(*ν*;*T*). The *χ*(*T*;*ν*) characteristics reveal that both compounds exhibit an SMM behaviour. That is, a maximum around 18 K in the out of phase component of the *χ*(*T*;*ν*) is observed for both SMMs at the highest frequency available of 1512 Hz and it shifts to lower temperatures as the frequency is decreased (see Fig. S7†). Noticeable differences between the two isotopologues are better seen in the frequency dependence of the susceptibility, thus we will first focus on these measurements.

**Fig. 5:**
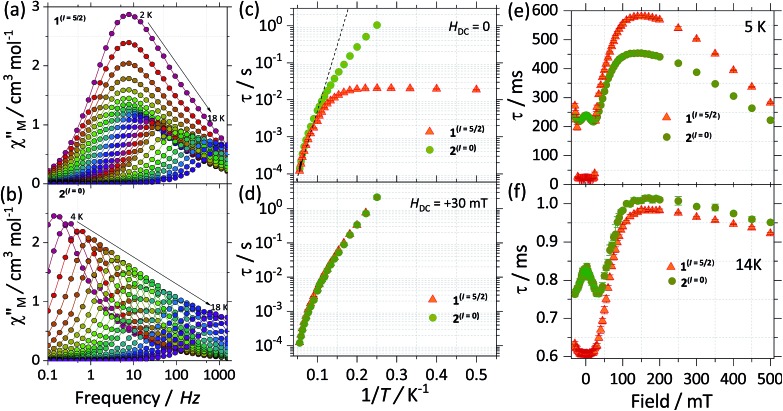
Experimental frequency dependent magnetic susceptibility data at zero applied DC (*H*_DC_) field and varied temperatures (*χ*_M_′′(*ν*)) for (a) **1**^(*I* = 5/2)^ and (b) **2**^(*I* = 0)^. Panel (c) and (d) shows the Arrhenius analysis for the *τ*(*T*) data for **1**^(*I* = 5/2)^ (orange) and **2**^(*I* = 0)^ (green), with the results obtained from fitting the *χ*_M_′′(*ν*) to a single Debye process at (c) *H*_DC_ = 0 and (d) *H*_DC_ = +30 mT. Field dependent study of the relaxation times (*τ*(*H*)) for **1**^(*I* = 5/2)^ (orange) and **2**^(*I* = 0)^ (green) at (a) 5 K and at (b) 14 K. *τ* were obtained after fitting the *χ*_M_′′(*ν*) to a single Debye process. The *τ*(*H*) data comprised field ranging between –30 mT to +500 mT and was collected with an oscillating field of 3.5 Oe.

Fig. 5a and b show the out of phase component of *χ*(*ν*;*T*) under a zero DC applied magnetic field for **1**^(*I* = 5/2)^ and **2**^(*I* = 0)^, respectively. For **1**^(*I* = 5/2)^, at the lowest temperature of 2 K, the maximum is centred around 7 Hz, and stays practically constant until reaching 5 K. Above 5 K the maximum in *χ*(*ν*;*T*) is clearly temperature dependent, shifting swiftly up to 18 K. In contrast, for the **2**^(*I* = 0)^ analogue, at the lowest temperature of 2 K, the maximum lies below our minimum working frequency of 0.1 Hz, while for temperatures between 4 K and 18 K the relaxation shows a strong temperature dependence. In order to compare the characteristic relaxation times of the two compounds at different temperatures we successfully fit the susceptibility measurements using the generalised Debye model: *χ*(*ν*) = *χ*_S_ + (*χ*_T_ – *χ*_S_)/(1 + (2*i*π*ν*)^1–*α*^), where *χ*_T_ and *χ*_S_ are the isothermal and adiabatic susceptibilities, respectively, *τ* is the relaxation time, and *α* indicates the distribution of relaxation times. The obtained temperature dependence of the relaxation times (*τ*(1/*T*)) is shown in Fig. 5c with the parameter *α* taking values between 0.02 < *α* < 0.37 for **1**^(*I* = 5/2)^, and 0.02 < *α* < 0.24 for **2**^(*I* = 0)^. The wide distribution of *α* and its decrease with temperature indicates the presence of multiple relaxation channels that affects the relaxation time (more so for **1**^(*I* = 5/2)^ than for the **2**^(*I* = 0)^ complex). The big difference between the relaxation time of **1**^(*I* = 5/2)^ and **2**^(*I* = 0)^ at low temperatures (*T* < 5 K) can be understood qualitatively by considering the effect of the nuclear spin on the processes that dominate the relaxation of the molecular spins in this temperature range.

First, for a polycrystalline sample, the presence of nuclear spins increases the fraction of molecules that can relax through quantum tunnelling. That is, the relaxation of **2**^(*I* = 0)^ through QTM takes place only when the bias local field satisfies the resonance condition (*H*_z_ ≈ *H*_r_), while for **1**^(*I* = 5/2)^ the hyperfine splitting leads to level anticrossings that are spread in the region of ±75 mT (Fig. 5b) and thus a larger fraction of molecules is found at resonance at any given time. Second, the hyperfine interaction in **1**^(*I* = 5/2)^ results in broader electronic levels and thus in a stronger coupling between the molecular spins and the vibrational acoustic modes (in a first approximation, the lifetime of an energy level is related to its width by the Heisenberg uncertainty principle, τ ∼ *ℏ*/Δ*E*). In the intermediate temperature range (2 K < *T* < 5 K), this leads to an increase in the rate of single phonon processes (direct relaxation) that dominates the spin–lattice relaxation dynamics. The stronger spin–phonon coupling for **1**^(*I* = 5/2)^ as compared to **2**^(*I* = 0)^ is also seen in the temperature dependence of the hysteresis loops obtained with the μ-SQUID technique (Fig. 3).

For temperatures larger than 5 K, the relaxation times of the two isotopologues are very close to each other and are well fitted by the Arrhenius law: *τ* = *τ*_0_ exp(–*U*_eff_/*k*_B_*T*). The fits shown in Fig. 5c lead to similar effective energy barriers: *U*_eff_ = 81.7(1) K for **1**^(*I* = 5/2)^ and *U*_eff_ = 81.0(1) K for **2**^(*I* = 0)^. As observed, the experimental *U*_eff_ is approximately half the separation between the ground state and first excited state obtained *via* CASSCF calculations, highlighting the importance of anharmonic phonons in the relaxation of complexes here studied.^22^

To investigate further the differences between the dynamic magnetic properties of the two isotopologues, *τ* was examined in detail by field dependent studies, *i.e. τ*(*H*) at a fixed temperature of 5 K with fields ranging from –30 mT to +500 mT (Fig. 5e). First, it should be noticed that the difference in the magnitude of the relaxation times of the two compounds is preserved for fields with an amplitude smaller than +25 mT. Also, for **2**^(*I* = 0)^, a modulation of *τ*(*H*) with a local maximum at zero and a minimum at around +30 mT is observed because when applying a small external field, the fraction of molecules that are found at resonance and can relax through QTM is increased. The polycrystalline nature of the sample is responsible for shifting the minimum to a smaller field value (*μ*_0_*H*_min_ ≈ 30 mT) as compared to the resonance field of about +35 mT observed for a monocrystal. At the same time, no such modulation is seen for **1**^(*I* = 5/2)^ because of the multiple hyperfine crossings and stronger spin–lattice coupling results in practically uniform relaxation rates. As we increase the field past +25 mT, a significant decrease in the relaxation rate is observed as the molecules are gradually shifted out of resonance and already at +30 mT the relaxation of the two compounds becomes very similar (Fig. 5d). At higher fields, *μ*_0_*H*_z_ > 200 mT, the relaxation is again enhanced due to the direct relaxation process (see also the μ-SQUID measurements in Fig. 3).

Interestingly, for fields larger than 100 mT the relaxation of **2**^(*I* = 0)^ is faster than that of **1**^(*I* = 5/2)^. This is unexpected and explaining it will require further investigations. However, in order to confirm that this is due to the presence/absence of nuclear spins we measured *τ*(*H*) at 14 K (Fig. 5f), where nuclear spin effects are expected to be less important and indeed the difference in *τ*(*H*) characteristics of the two isotopologues is greatly reduced, thus nuclear spin effects are of less relevance at higher temperatures.

## Conclusions

Two dinuclear ^163/164^Dy isotopologues have been synthesised and structurally and magnetically characterised. Both complexes are SMMs, however, they show marked differences in the dynamic properties as revealed by AC and μ-SQUID studies at sub-Kelvin temperatures. μ-SQUID loops reveal an interaction between the two Dy(iii) ions, leading to S-shaped hysteresis loops characteristic of antiferromagnetically coupled Ising spins. A closer inspection of the temperature dependent hysteresis loops shows that the relaxation is slower in **2**^(*I* = 0)^ than in **1**^(*I* = 5/2)^. This can be mainly ascribed to the absence of nuclear spins in **2**^(*I* = 0)^. Fitting the hysteresis loops reveals that the tunnelling rate in both complexes is equal, therefore, tunnelling does not solely play a role in the relaxation dynamic of both complexes. Instead, the larger spectrum of hyperfine states in **1**^(*I* = 5/2)^ allows a better coupling to acoustic phonons, consequently enhancing the direct relaxation process at sub-Kelvin temperatures compared to **2**^(*I* = 0)^, possessing no hyperfine states. Note that phonons modulate the electric field of the magnetic ions, therefore inducing direct relaxation process.^20^,^23^ Now, if we consider **2**^(*I* = 0)^, the absence of nuclear spins states leads to a ground doublet state with no hyperfine-split levels. In contrast, in **1**^(*I* = 5/2)^ the hyperfine level splits the electronic state in (2*I* + 1)^2^ states, thus a total of 36 states (for *I* = 5/2) comprise the ground doublet. At very low temperatures, where direct relaxation is important, the number of available phonon modes with energy corresponding to the difference between the spins state is therefore larger for **1**^(*I* = 5/2)^ than for **2**^(*I* = 0)^.

The difference in the relaxation rate of the two compounds, at low temperatures and small magnetic fields, is also clearly evidenced in AC measurements. Note that, the nuclear spin effects are more important at lower temperatures than at higher as revealed by the *τ*(*H*) at 5 and 14 K. In both Dy_2_ isotopologues here studied, we show that the tunnelling probabilities are not affected by the nuclear spins and play a minor role in well-performing SMMs, where the operating temperatures are rather large, in agreement with recent studies.^11^ Finally, we argue that although we and others find that tunnelling is not affected by the nuclear spin presence/absence, the hyperfine level broadening still plays an important role for SMMs with moderate energy barriers (more importantly at very low temperatures), since it facilitates the spin–phonon coupling, thus enhancing the direct relaxation process. These finding must be contemplated for Ln-SMMs proposed for very low temperature applications, such as quantum bits, where the utilisation of the nuclear states embedded in the lanthanide ions can be used as quantum bits. For example, the indirect coupling of the nuclear states, *via* the electronic states, would increase the number of nuclear states available for the realisation of complex quantum algorithms.^24^

## Conflicts of interest

There are no conflicts to declare.

## Supplementary Material

Supplementary information

Crystal structure data

## References

[cit1] Gatteschi D., Sessoli R., Villain J., Molecular nanomagnets, Oxford University Press, Oxford, UK, 2006.

[cit2] Liddle S. T., van Slageren J. (2015). Chem. Soc. Rev..

[cit3] Moreno-Pineda E., Godfrin C., Balestro F., Wernsdorfer W., Ruben M. (2018). Chem. Soc. Rev..

[cit4] Godfrin C., Ferhat A., Ballou R., Klyatskaya S., Ruben M., Wernsdorfer W., Balestro F. (2017). Phys. Rev. Lett..

[cit5] Gregson M., Chilton N. F., Ariciu A.-M., Tuna F., Crowe I. F., Lewis W., Blake A. J., Collison D., McInnes E. J. L., Winpenny R. E. P., Liddle S. T. (2016). Chem. Sci..

[cit6] Woodruff D. N., Winpenny R. E. P., Layfield R. A. (2013). Chem. Rev..

[cit7] Chen Y.-C., Liu J.-L., Ungur L., Liu J., Li Q.-W., Wang L.-F., Ni S.-P., Chibotaru L. F., Chen S.-M., Tong M.-L. (2016). J. Am. Chem. Soc..

[cit8] Luo M., Wang X. (2014). Sci. China: Phys., Mech. Astron..

[cit9] Gatteschi D., Sessoli R. (2003). Angew. Chem., Int. Ed..

[cit10] Tesi L., Salman S., Cimatti I., Pointillart F., Bernot K., Mannini M., Sessoli R. (2018). Chem. Sci..

[cit11] (b) Ortu F., Reta D., Ding Y.-S., Goodwin C. A. P., Gregson M. P., McInnes E. J. L., Winpenny R. E. P., Sheng Y.-S., Liddle S. T., Mills D. P. and Chilton N. F., ChemRxiv, 2018, 10.26434/chemrxiv.6790568.v1. 31112169

[cit12] Yu W., Schramm F., Moreno-Pineda E., Lan Y., Fuhr O., Chen J., Isshiki H., Wernsdorfer W., Wulfhekel W., Ruben M. (2016). Beilstein J. Nanotechnol..

[cit13] Alvares S., Alemany P., Casanova D., Cirera J., Llunell M., Avnir D. (2005). Coord. Chem. Rev..

[cit14] Wernsdorfer W., Chakov N. E., Christou G. (2004). Phys. Rev. B: Condens. Matter Mater. Phys..

[cit15] Moreno-Pineda E., Lan Y., Fuhr O., Wernsdorfer W., Ruben M. (2017). Chem. Sci..

[cit16] Aquilante F., Autschbach J., Carlson R. K., Chibotaru L. F., Delcey M. G., De Vico L., Galván I. F., Ferré N., Frutos L. M., Gagliardi L., Garavelli M., Giussani A., Hoyer C. E., Li Manni G., Lischka H., Ma D., Malmqvist P. Å., Müller T., Nenov A., Olivucci M., Pedersen T. B., Peng D., Plasser F., Pritchard B., Reiher M., Rivalta I., Schapiro I., Segarra-Martí J., Stenrup M., Truhlar D. G., Ungur L., Valentini A., Vancoillie S., Veryasov V., Vysotskiy V. P., Weingart O., Sapata F., Lindh R. (2016). J. Comput. Chem..

[cit17] Siegbahn P. E. M., Almlçf J., Heiberg A., Roos B. O. (1981). J. Chem. Phys..

[cit18] Malmqvist P.-Å., Roos B. O., Schimmelpfennig B. (2002). Chem. Phys. Lett..

[cit19] Ungur L., Chibotaru L. F. (2017). Chem.–Eur. J..

[cit20] Abragam A., Bleaney B., Electron Paramagnetic Resonance of Transition Ions, Dover, New York, 1986, ch. 10.

[cit21] Culvahouse J. W., Unruh W. P., Brice D. (1963). Phys. Rev..

[cit22] Lunghi A., Totti F., Sessoli R., Sanvito S. (2016). Nat. Commun..

[cit23] Ganzhorn M., Klyatskaya S., Ruben M., Wernsdorfer W. (2013). Nat. Nanotechnol..

[cit24] Gaita-Ariño A., Luis F., Hill S., Coronado E. (2019). Nat. Chem..

[cit25] Sheldrick G. M. (2008). Acta Crystallogr., Sect. A: Found. Crystallogr..

[cit26] Dolomanov O. V., Bourthis L. J., Gildea R. L., Howard J. A. K., Puschmann H. (2009). J. Appl. Crystallogr..

