# Supplementary material for: Quantum tunnelling of the magnetisation in single-molecule magnet isotopologue dimers
Source: Chem Sci. 2019 Apr 15;10(19):5138–45. doi: 10.1039/c9sc01062a (PMC6524608; doi:10.1039/c9sc01062a)
Supplement: Supplementary file 1 [file SC-010-C9SC01062A-s001.pdf]

Electronic Supplementary Material (ESI) for Chemical Science.
This journal is © The Royal Society of Chemistry 2019

Supporting Information

Quantum Tunnelling of the Magnetisation in Single-Molecule Magnet Isotopologue Dimers

Eufemio Moreno-Pineda[*a], Gheorghe Taran[b], Wolfgang Wernsdorfer[*abc] and Mario Ruben[*ad]*

a. Institute of Nanotechnology (INT), Karlsruhe Institute of Technology (KIT), Hermann-von-Helmholtz-Platz 1, D-76344 Eggenstein-Leopoldshafen, Germany. E-mail: eufemio.pineda@kit.edu; wolfgang.wernsdorfer@kit.edu; mario.ruben@kit.edu.
b. Physikalisches Institut, Karlsruhe Institute of Technology, D-76131 Karlsruhe, Germany
c. CNRS, Institut Néel, F-38042 Grenoble, France.
d. Institut de Physique et Chimie des Matériaux de Strasbourg (IPCMS), CNRS-Université de Strasbourg, 23 rue du Loess, BP 43, F-67034 Strasbourg Cedex 2, France.

A. Experimental Details

Synthetic Method

Solvents and reagents were of commercial grade and used without further purification. A mixture of the respective Ln(tmhd)$_3$·(H$_2$O)$_2$ [Ln = ^{163}Dy (III), $\mathbf{1}^{(I\,=\,5/2)}$ and ^{164}Dy(III), $\mathbf{2}^{(I\,=\,0)}$] (1 mmol) precursor and bpym (0.5 mmol) was stirred in absolute ethanol (20 mL) for 24 hours. The precipitated obtained was subsequently crystallised from a mixture of EtOH and Et$_2$O at 5 ºC. Elemental analysis: for $Dy_2O_{12}N_4C_{74}H_{120}$ ($\mathbf{1}^{(I\,=\,5/2)}$) calculated (found) (%): C 56.15 (56.02), H 7.64 (7.37), N, 3.54 (3.49); for $Dy_2O_{12}N_4C_{74}H_{120}\cdot OC_4H_{10}$ ($\mathbf{2}^{(I\,=\,0)}$) calculated (found) (%): C 56.15 (55.92), H 7.64 (7.67, N, 3.54 (3.53)

Crystallography

Crystallographic data for $\mathbf{1}^{(I\,=\,5/2)}$ [$C_{78}H_{130}Dy_2N_4O_{14}$]: Mr = 1672.85, triclinic, T = 180.0(2) K, a = 10.8743(2), b = 13.8790(3), c = 14.8618 (4) Å, α = 90.039(2)º, β = 93.333(2)º, γ = 107.553(2)º, V = 2131.63(9) Å^3, Z = 1, ρ = 1.303 g cm^{-3}, total data = 25289, independent reflections 8697 (R_{int} = 0.0289), μ = 1.797 mm^{-1}, 467 parameters, R_1 = 0.0256 for I ≥2σ (I) and wR_2 = 0.0630. Crystal data for $\mathbf{2}^{(I\,=\,0)}$ [$C_{78}H_{130}Dy_2N_4O_{13}$]: Mr = 1672.85, triclinic, T = 180.0(2) K, a = 10.8671(2), b = 13.8736(3), c = 14.8582(3) Å, α = 91.995(2)º, β = 93.347(2)º, γ = 107.530(2)º, V = 2129.20(8) Å^3, Z = 1, ρ = 1.305 g cm^{-3}, total data = 24512, independent reflections 8663 (R_{int} = 0.0325), μ = 1.797 mm^{-1}, 480 parameters, R_1 = 0.0241 for I ≥2σ (I) and wR_2 = 0.0610. Single crystal X-ray diffraction data of $\mathbf{1}^{(I\,=\,5/2)}$ and $\mathbf{2}^{(I\,=\,0)}$ was collected employing a STOE StadiVari 25 diffractometer with a Pilatus300 K detector using GeniX 3D HF micro focus with MoKα radiation (λ = 0.71073 Å). The structure was solved using direct methods and was refined by full-matrix least-squares methods on all F_2 using SHELX-2014[1] implemented in Olex2[2]. The

1 [1] G. M. Sheldrick, *Acta Crystallogr., Sect. A: Found. Crystallogr.*, 2008, **64**, 112.
2 [2] O. V. Dolomanov, L. J. Bourthis, R. L. Gildea, J. A. K.Howard and H. Puschmann, *J. Appl. Crystallogr.*, 2009, **42**, 339.

crystals were mounted on a glass tip using crystallographic oil and placed in a cryostream. Data were collected using φ and ω scans chosen to give a complete asymmetric unit. All non-hydrogen atoms were refined anisotropically. Hydrogen atoms were calculated geometrically riding on their parent atoms. Full crystallographic details can be found in CIF format: see the Cambridge Crystallographic Data Centre database (CCDC 1898383 and 1898384 for $\mathbf{1}^{(I\,=\,5/2)}$ and $\mathbf{2}^{(I\,=\,0)}$, respectively).

SQUID data

Magnetic susceptibility measurements were collected using Quantum Design MPMS-XL SQUID magnetometers on polycrystalline material in the temperature range 2 – 300 K under an applied DC magnetic field (H_{DC}) of 1 kOe. AC data was collected using an oscillating magnetic field of 3.5 Oe and frequencies between 0.1 and 1.5 kHz. DC data were corrected for diamagnetic contributions from the eicosane and core diamagnetism employing Pascal's constants. Low temperature (0.03 – 5 K) magnetisation measurements were performed on single crystals using a μ-SQUID apparatus at different sweep rates between 0.280 and 0.002 T s^{-1}. The time resolution is approximately 1 ms. The magnetic field can be applied in any direction of the micro-SQUID plane with precision much better than 0.1° by separately driving three orthogonal coils. In order to ensure good thermalisation, each sample was fixed with apiezon grease.

CASSCF Calculation

For the CASSCF-SO electronic structure calculation of compounds $\mathbf{1}^{(I\,=\,5/2)}$ and $\mathbf{2}^{(I\,=\,0)}$ we employed MOLCAS 8.2.[3,4] Due to the symmetry consideration, CASSCF-SO calculation was performed only on a single Dy(III) site. For this, solely a single $Dy(tmhd)_3$ ion attached to the bypm fragment was employed, i.e. ($Dy(tmhd)_3$bypm), using the crystallographic coordinated obtained from the single crystal X-ray structure with no further optimisation. Basis sets from ANO-RCC library[5] were employed with VTZP quality for Dy, VDZP quality for the bipyrimidine and for all O of the tmhd groups, while VDZ quality for all remaining atoms was employed, using the second-order DKH transformation.[6] The molecular orbitals (MOs) were optimized in state-averaged CASSCF calculations. For this, the active space was defined by the nine 4f

[3] F. Aquilante, J. Autschbach, R. K. Carlson, L. F. Chibotaru, M. G. Delcey, L. De Vico, I. Fdes. Galván, N. Ferré, L. M. Frutos, L. Gagliardi, M. Garavelli, A. Giussani, C. E. Hoyer, G. Li Manni, H. Lischka, D. Ma, P. Å. Malmqvist, T. Müller, A. Nenov, M. Olivucci, T. B. Pedersen, D. Peng, F. Plasser, B. Pritchard, M. Reiher, I. Rivalta, I. Schapiro, J. Segarra-Martí, M. Stenrup, D. G. Truhlar, L. Ungur, A. Valentini, S. Vancoillie, V. Veryasov, V. P. Vysotskiy, O. Weingart, F. Sapata and R. Lindh, *J. Comput. Chem.*, 2016, **37**, 506–541.
[4] (a) P. E. M. Siegbahn, J. Almlçf, A. Heiberg, B.O.Roos, J. Chem. Phys. 1981, **74**, 2384–2396; (b) J. Olsen, B. O. Roos, P. Jørgensen, H. J. A. Jensen, *J. Chem. Phys*. 1988, **89**, 2185–2192.
[5] (a) B. O. Roos, R.Lindh, P.-Å.Malmqvist, V.Veryazov and P.-O.Widmark, *J. Phys. Chem. A*. 2004, **108**, 2851–2858; (b) B. O. Roos, R. Lindh, P.-A. Malmqvist, V. Veryazov, P.-O. Widmark, *J. Phys. Chem. A*, 2008, **112**, 11431-11435; (c) P.-O. Widmark, P.-A. Malmqvist, and B. O. Roos, *Theor. Chim. Acta* 1990, **77**, 291.
[6] D. Peng and K. Hirao, *J. Chem. Phys.*, 2009, **130**, 044102.

electrons in the seven 4f orbitals of Dy(III). Three calculations were performed independently for each possible spin state, where 21 roots were included for *S* = 5/2, 224 roots were included for *S* = 3/2, and 490 roots were for *S* = ½ (RASSCF routine). The wavefunctions obtained from these CASSCF calculations were posteriorly mixed by spin orbit coupling, where all 21 *S* = 5/2 states, 128 of the *S* = 3/2 states, and 130 of the *S* = 1/2 states were included (RASSI routine[7]). The resulting spin orbit wavefunctions were decomposed into their CF wavefunctions in the $^6H_{15/2}$ basis, employing the SINGLE_ANISO[8] routine, and the magnetic susceptibility was calculated.

The simulations within Ising model was carried out employing an Exchange Ising Hamiltonian of the following form:

$$\hat{H}_{ex} = J_{total}(\hat{s}_{1,z} + \hat{s}_{2,z}) \quad \text{(S1)}$$

where $\hat{s}_{i,z}$ is the projection of the pseudo spins $\tilde{s} = 1/2$ on the main axes of the centre *i*. J_{total} is the total exchange interaction comprising the exchange interaction (J_{ex}) and the dipolar interaction (J_{dip}) between the centres, i.e. $J_{total} = J_{ex} + J_{dip}$.

The dipolar interaction can be determined employing eq. S2 and the parameters determined from ab-initio calculations.

$$J_{dip} = \frac{\mu_B^2}{r^3} - \left[\bar{\bar{g}}_A \cdot \bar{\bar{g}}_B - 3\left(\bar{\bar{g}}_A \cdot \vec{R}\right) \cdot \left(\vec{R}^T \cdot \bar{\bar{g}}_B\right)\right] \quad \text{(S2)}$$

[7] P.-Å. Malmqvist, B. O. Roos, B. Schimmelpfennig, *Chem. Phys. Lett.* 2002, **357**, 230–240.
[8] (a) L. Ungur, L. F. Chibotaru, *Chem. Eur. J.*, 2017, **23**, 3708–3718; (b) L. F. Chibotaru and L. Ungur, *J. Chem. Phys.*, 2012, **137**, 064112.

B. Supplementary Tables

Crystallographic Tables

Table S1. Crystallographic information for clusters $\mathbf{1}^{(I\,=\,5/2)}$ and $\mathbf{2}^{(I\,=\,0)}$.

	$\mathbf{1}^{(I\,=\,5/2)}$	$\mathbf{2}^{(I\,=\,0)}$
formula	$Dy_2N_4O_{14}C_{78}H_{130}$	$C_{76}H_{125}Dy_2N_4O_{13}$
FW / g mol^{-1}	1672.85	1627.79
crystal system	triclinic	triclinic
space group	*P-1*	*P-1*
a/Å	10.8743(2)	10.8671(2)
b/Å	13.8790(3)	13.8736(3)
c/Å	14.8618(4)	14.8582(3)
α/°	92.039(2)	91.995(2)
β/°	93.333(2)	93.347(2)
γ/°	107.553(2)	107.530(2)
V/Å^3	2131.63(9)	2129.70(8)
Z	1	1
ρ calcd/g cm^{-3}	1.303	1.305
T/K	180.15	180.15
μ (Mo K_α)/mm^{-1}	1.797	1.797
$R_1(I>2\sigma)(I))^a$	0.0256	0.0241
wR_2^a	0.0630	0.0610

^{a} $R_1 = ||F_0| - |F_c||/|F_0|$, $wR_2 = [w(|F_0| - |F_c|)^2/w|F_0|^2]^{1/2}$

Table S2. Continuous shaped measures (CShM) for Dy(III) ions in compounds $\mathbf{1}^{(I\,=\,5/2)}$ and $\mathbf{2}^{(I\,=\,0)}$.

	$\mathbf{1}^{(I\,=\,5/2)}$	$\mathbf{2}^{(I\,=\,0)}$
**OP-8**	30.246	30.233
**HPY-8**	22.474	22.398
**HBPY-8**	15.434	15.425
**CU-8**	8.808	8.807
**SAPR-8**	0.607	0.615
**TDD-8**	2.375	2.365
**JGBF-8**	16.142	16.093
**JETBPY-8**	27.616	27.600

OP-8 = (D_{8h}) Octagon
HPY-8 = (C_{7v}) Heptagonal pyramid
HBPY-8 = (D_{6h}) Hexagonal bipyramid
CU-8 = (*Oh*) Cube
SAPR-8 = (D_{4d}) Square antiprism
TDD-8 = (D_{2d}) Triangular dodecahedron
JGBF-8 = (D_{2d}) Johnson gyrobifastigium J26
JETBPY-8 = (D_{3h}) Johnson elongated triangular bipyramid J14

Table S3. Electronic structure of (Dy(tmhd)$_3$bypm) calculated with CASSCF-SO using solid-state geometry from $\mathbf{1}^{(I = 5/2)}$.

Energy (cm^{-1})	Energy (K)	g_x	g_x	g_x	Angle (º)
0.0	0.0	0.0019	0.0035	19.3476	-
130.1	187.2	0.2289	0.2986	15.4042	21.8
186.3	278.1	1.3556	2.0760	12.7208	23.1
218.7	314.7	3.4716	5.1106	9.8219	137.4
264.0	379.9	2.0218	2.9504	9.3195	57.1
303.6	436.8	0.7811	1.7781	16.06935	111.2
434.0	624.5	0.0214	0.0327	17.0889	78.4
507.9	730.9	0.0073	0.0214	18.5472	56.7

The ground state wavefunction has a composition of: 98.9%$|\pm 15/2\rangle$, 0.9%$|\pm 11/2\rangle$ and 0.2% $|\pm 13/2\rangle$.

Table S4. Electronic structure of (Dy(tmhd)$_3$bypm) calculated with CASSCF-SO using solid-state geometry from $\mathbf{2}^{(I = 0)}$.

Energy (cm^{-1})	Energy (K)	g_x	g_x	g_x	Angle (º)
0.0	0.0	0.0014	0.0032	19.369	-
130.8	188.2	0.2408	0.3047	15.4117	21.2
185.8	267.3	1.4521	2.2595	12.8100	24.8
217.2	312.4	3.3381	5.1836	9.5849	137.7
263.1	378.5	2.1595	2.9306	9.3460	56.8
303.1	436.0	0.7294	1.6570	15.9936	111.0
434.7	625.4	0.0186	0.0355	17.0662	78.2
507.7	730.4	0.0084	0.0242	18.5192	56.7

The ground state wavefunction has a composition of: 99.3%$|\pm 15/2\rangle$, 0.3%$|\pm 11/2\rangle$ and 0.3% $|\pm 13/2\rangle$.

Table S5. Percentage composition of the lowest multiplet J = 15/2 for $\mathbf{1}^{(I = 5/2)}$

m_J	Energy (K) of KD															
	0		187.2		278.1		314.7		379.9		436.8		624.5		730.9	
$\lvert -15/2\rangle$	0.0	98.9	0.0	0.2	0.0	0.7	0.0	0.0	0.0	0.2	0.0	0.0	0.0	0.0	0.0	0.0
$\lvert -13/2\rangle$	0.0	0.1	0.0	88.7	0.1	0.1	0.7	7.5	1.2	0.2	1.1	0.0	0.1	0.1	0.0	0.0
$\lvert -11/2\rangle$	0.0	0.9	0.0	0.7	0.1	57.7	6.6	1.3	4.0	20.0	0.9	6.1	1.3	0.4	0.0	0.0
$\lvert -9/2\rangle$	0.0	0.0	0.0	9.2	0.2	0.2	2.0	36.2	9.7	9.8	21.3	4.1	0.5	6.4	0.4	0.0
$\lvert -7/2\rangle$	0.0	0.0	0.0	0.1	0.1	29.3	0.0	0.1	4.4	15.5	8.0	20.5	18.2	1.4	0.3	2.1
$\lvert -5/2\rangle$	0.0	0.0	0.0	1.0	0.7	1.8	1.1	30.5	0.0	0.3	18.3	3.0	1.8	30.8	9.4	1.3
$\lvert -3/2\rangle$	0.0	0.0	0.0	0.0	0.1	7.0	3.5	0.1	4.8	20.9	0.0	0.3	28.8	3.9	2.8	27.8
$\lvert -1/2\rangle$	0.0	0.0	0.0	0.1	1.6	0.4	0.2	10.2	7.4	1.7	14.5	1.8	0.9	5.3	52.8	3.1
$\lvert +1/2\rangle$	0.0	0.0	0.1	0.0	0.4	1.6	10.2	0.2	1.7	7.4	1.8	14.5	5.3	0.9	3.1	52.8
$\lvert +3/2\rangle$	0.0	0.0	0.0	0.0	7.0	0.1	0.1	3.5	20.9	4.8	0.3	0.0	3.9	28.8	27.8	2.8
$\lvert +5/2\rangle$	0.0	0.0	1.0	0.0	1.8	0.7	30.5	1.1	0.3	0.0	3.0	18.3	30.8	1.8	1.3	9.4
$\lvert +7/2\rangle$	0.0	0.0	0.1	0.0	29.3	0.1	0.1	0.0	15.5	4.4	20.5	8.0	1.4	18.2	2.1	0.3
$\lvert +9/2\rangle$	0.0	0.0	9.2	0.0	0.2	0.2	36.2	2.0	9.8	9.7	4.1	21.3	6.4	0.5	0.0	0.4
$\lvert +11/2\rangle$	0.9	0.0	0.7	0.0	57.7	0.1	1.3	6.6	20.0	4.0	6.1	0.9	0.4	1.3	0.0	0.0
$\lvert +13/2\rangle$	0.1	0.0	88.7	0.0	0.1	0.1	7.5	0.7	0.2	1.2	0.0	1.1	0.1	0.1	0.0	0.0
$\lvert +15/2\rangle$	98.9	0.0	0.2	0.0	0.7	0.0	0.0	0.0	0.2	0.0	0.0	0.0	0.0	0.0	0.0	0.0

Table S6. Percentage composition of the lowest multiplet J = 15/2 for $\mathbf{2}^{(I=0)}$.

m_J	Energy (K) of KD															
	**0**		**187.2**		**278.1**		**314.7**		**379.9**		**436.8**		**624.5**		**730.9**	
$\|-15/2\rangle$	0.0	98.9	0.0	0.2	0.6	0.0	0.0	0.0	0.0	0.1	0.0	0.0	0.0	0.0	0.0	0.0
$\|-13/2\rangle$	0.0	0.2	0.0	89.8	0.2	0.1	0.1	7.3	1.2	0.0	0.9	0.1	0.1	0.0	0.0	0.0
$\|-11/2\rangle$	0.0	0.8	0.0	0.9	60.0	0.0	3.0	3.4	1.1	22.2	2.3	4.7	0.7	0.8	0.0	0.0
$\|-9/2\rangle$	0.0	0.0	0.0	8.1	0.1	0.2	1.0	38.5	13.1	6.2	15.0	10.6	2.1	4.9	0.1	0.1
$\|-7/2\rangle$	0.0	0.0	0.0	0.0	28.0	0.0	0.0	0.0	1.9	18.6	16.3	13.2	14.5	5.4	0.5	1.5
$\|-5/2\rangle$	0.0	0.0	0.9	1.5	0.5	1.4	30.1	0.0	0.4	12.9	8.5	8.0	25.3	7.7	2.8	2.8
$\|-3/2\rangle$	0.0	0.0	0.0	0.0	6.9	0.0	2.4	1.3	1.9	23.7	0.1	0.4	21.5	10.8	9.2	21.7
$\|-1/2\rangle$	0.0	0.0	0.0	0.1	0.3	1.5	0.8	10.5	8.9	0.6	9.6	5.4	2.2	3.8	36.3	20.0
$\|+1/2\rangle$	0.0	0.0	0.1	0.0	1.5	0.3	10.5	0.8	0.6	8.9	5.4	9.6	3.8	2.2	20.0	36.3
$\|+3/2\rangle$	0.0	0.0	0.0	0.0	0.0	6.9	1.3	2.4	23.7	1.9	0.4	0.1	10.8	21.5	21.7	9.2
$\|+5/2\rangle$	0.0	0.0	0.9	0.5	1.5	30.1	1.4	0.4	0.0	8.5	12.9	25.3	8.0	2.8	7.7	2.8
$\|+7/2\rangle$	0.0	0.0	0.0	0.0	0.0	28.0	0.0	0.0	18.6	1.9	13.2	16.3	5.4	14.5	1.5	0.5
$\|+9/2\rangle$	0.0	0.0	8.1	0.0	0.2	0.1	38.5	1.0	6.2	13.1	10.6	15.0	4.9	2.1	0.1	0.1
$\|+11/2\rangle$	0.8	0.0	0.9	0.0	0.0	60.0	3.4	3.0	22.2	1.1	4.7	2.3	0.8	0.7	0.0	0.0
$\|+13/2\rangle$	0.2	0.0	89.8	0.0	0.1	0.2	7.3	0.1	0.0	1.2	0.1	0.9	0.0	0.1	0.0	0.0
$\|+15/2\rangle$	98.9	0.0	0.2	0.0	0.0	0.6	0.0	0.0	0.1	0.0	0.0	0.0	0.0	0.0	0.0	0.0

Supplementary Figures

1. IR.

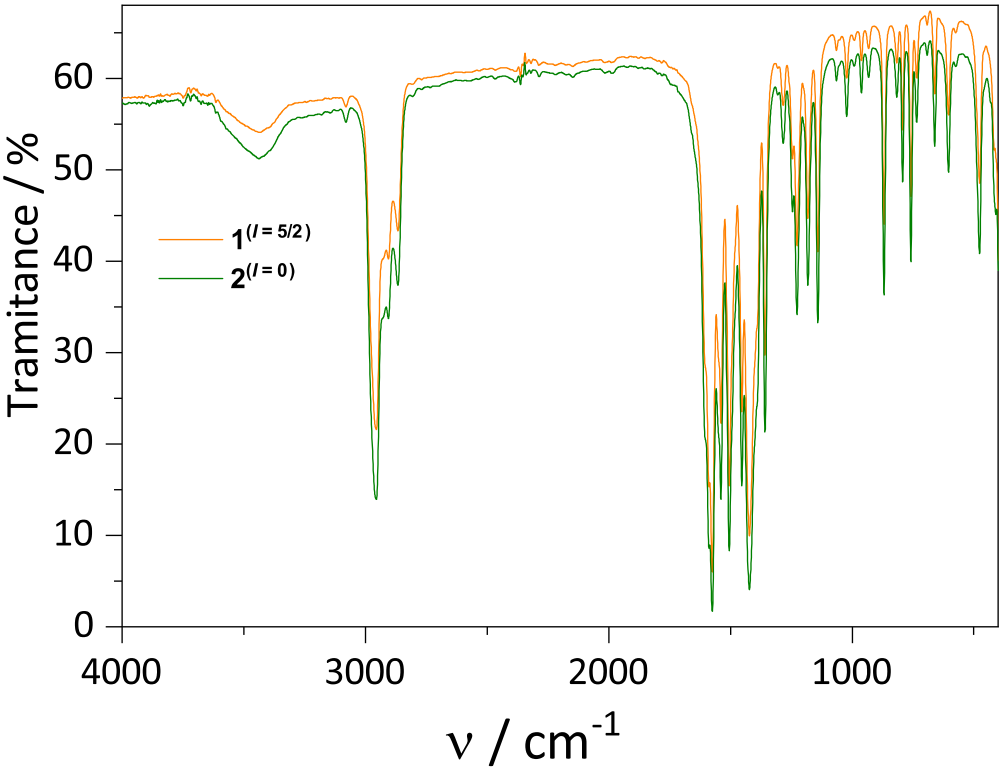

Figure S1. Infrared spectra for compound **1**$^{(I = 5/2)}$ (blue) and **2**$^{(I = 0)}$ (green).

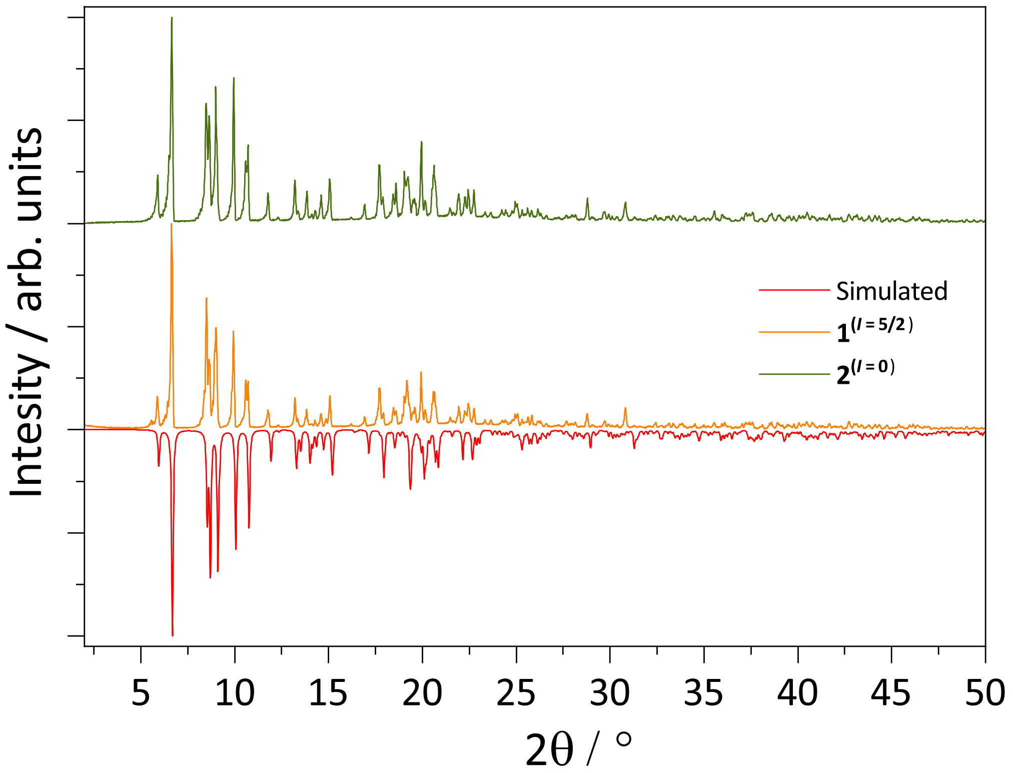

Figure S2. Simulated powder diffraction patter (red) and experimental patterns for complexes $\mathbf{1}^{(I=5/2)}$ (orange) and $\mathbf{2}^{(I=0)}$ (green).

CASSCF-SO Calculations

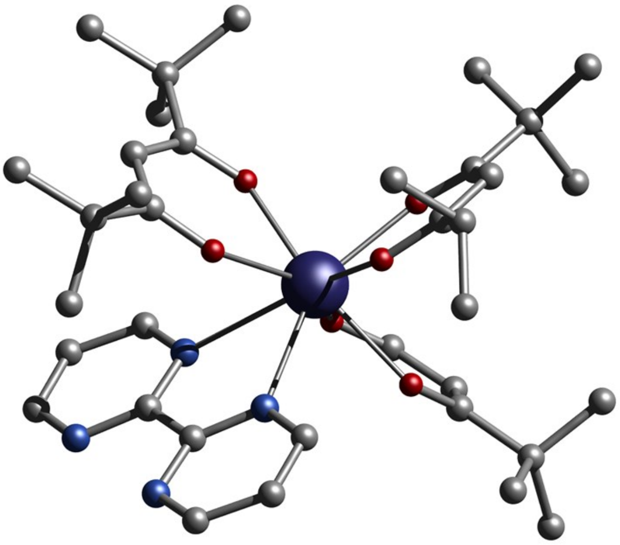

Figure S3. Molecular fragment employed for the CASSCF-SO calculations.

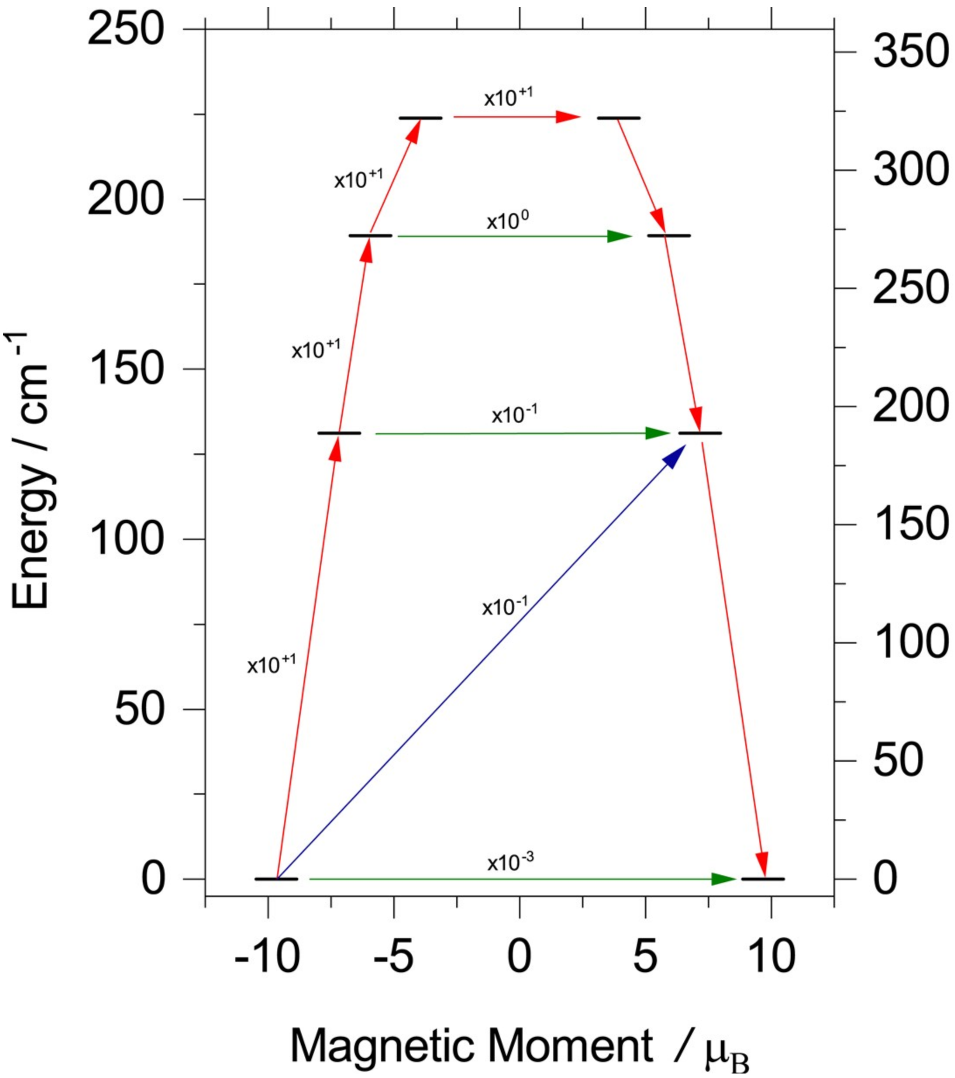

Figure S4. Relaxation of the magnetisation in **1**$^{(I = 5/2)}$ and **2**$^{(I = 0)}$. Red arrows show the most probable relaxation route and light green and blue arrows indicate less significant but non-negligible matrix elements between states.

2. Magnetism

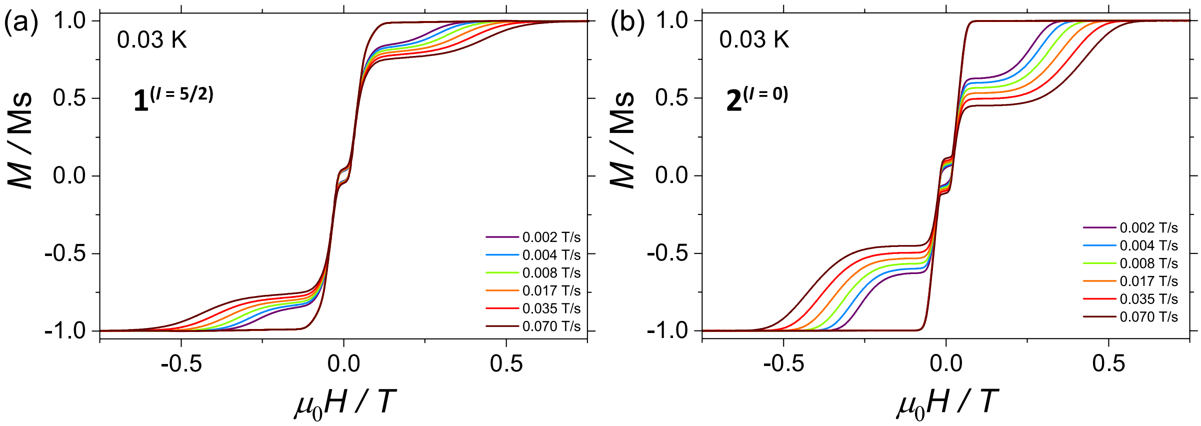

Figure S5. μ-SQUID data of $\mathbf{1}^{(I = 5/2)}$ (a) and $\mathbf{2}^{(I = 0)}$ obtained at different field sweep rates at (a) 0.02 K.

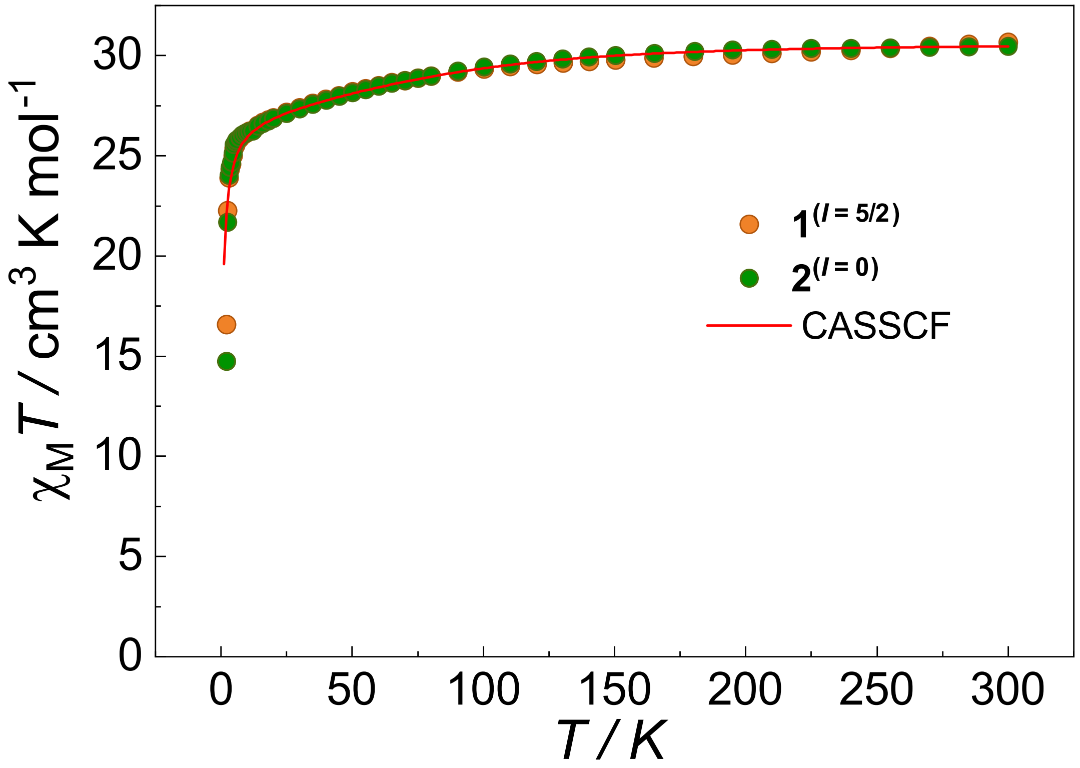

Figure S6. Experimental $\chi_M T$ for $\mathbf{1}^{(I = 5/2)}$ (orange circles) and $\mathbf{2}^{(I = 0)}$ (green circles) and $\chi_M T$ obtained employing the crystal field parameters obtained using the SINGLE_ANISO module after CASSCF-SO calculations and an isotropic interaction operating between the spin component of the angular momenta of the Dy(III) ions ($S = 5/2$), as determined from μ-SQUID data ($J_{total} = J_{ex} + J_{dip}$). The Simulation corresponds to $J_{total} = 4.18$ mK, $J_{dip} = 3.30$ mK and $J_{ex} = 0.88$ mK. See CASSCF section details. Note that a scaling factor of 1.08 was applied to the simulated $\chi_M T$ data.

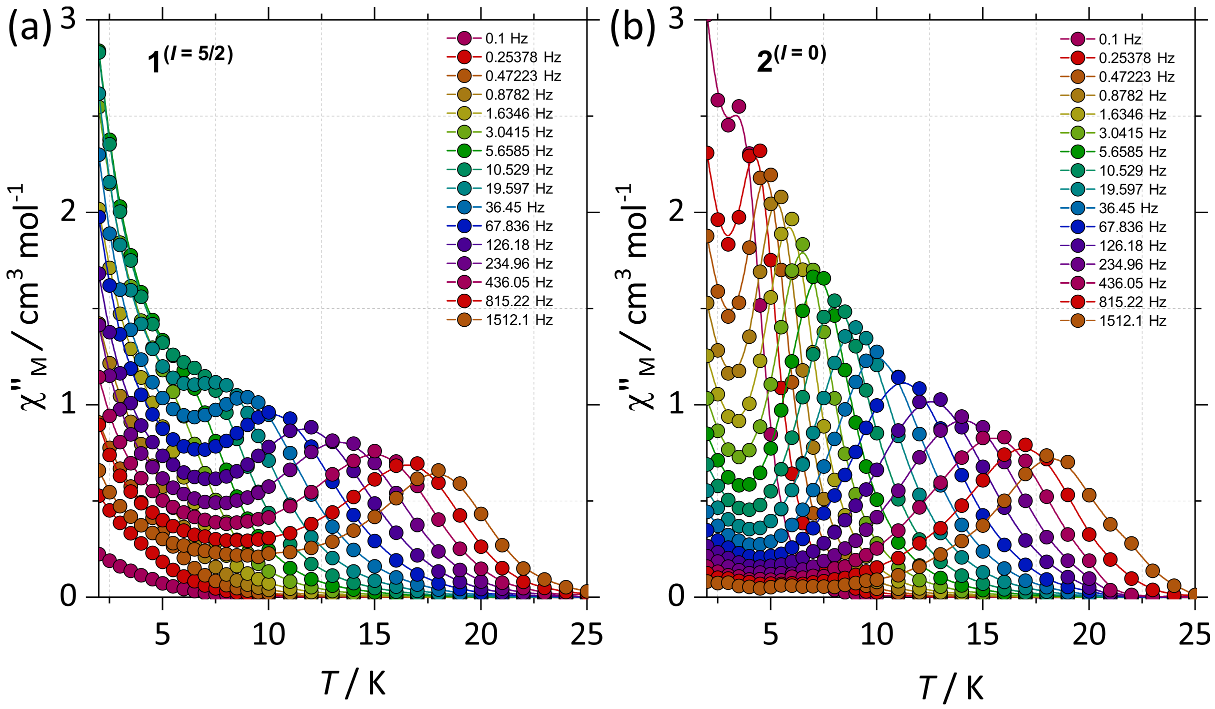

Figure S7. Temperature dependent AC magnetic data for compounds (a) $\mathbf{1}^{(I = 5/2)}$ and (b) $\mathbf{1}^{(I = 5/2)}$ employing a 3.5 Oe AC field and H_{DC} = 0. Solid lines are guide to the eye.

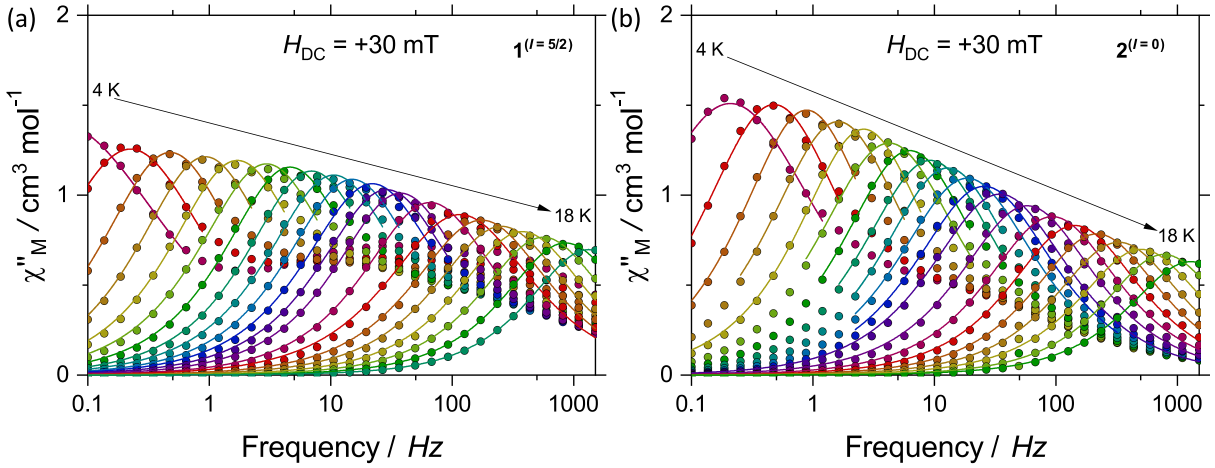

Figure S8. Experimental frequency dependent magnetic susceptibility data with H_{DC} = +30 mT applied field at varied temperatures ($\chi_M''(\nu)$) for (a) $\mathbf{1}^{(I=5/2)}$ and (b) $\mathbf{2}^{(I=0)}$. Solid lines are best fit to a single Debye process.

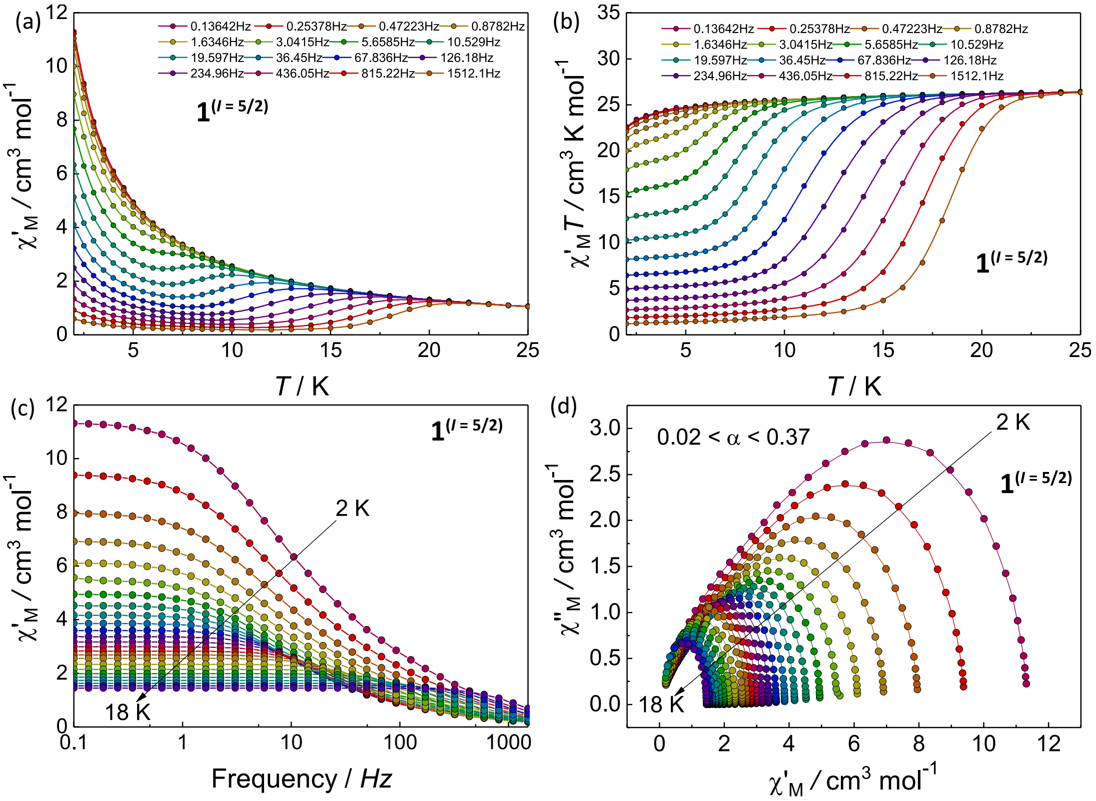

Figure S9. Dynamic magnetic data for compound $\mathbf{1}^{(I=5/2)}$ employing a 3.5 Oe AC field and zero DC field. (a) $\chi_M'(T)$;(b) $\chi_M''T(T)$; (c) $\chi_M'(\nu)$ and (d) Cole-Cole plots.

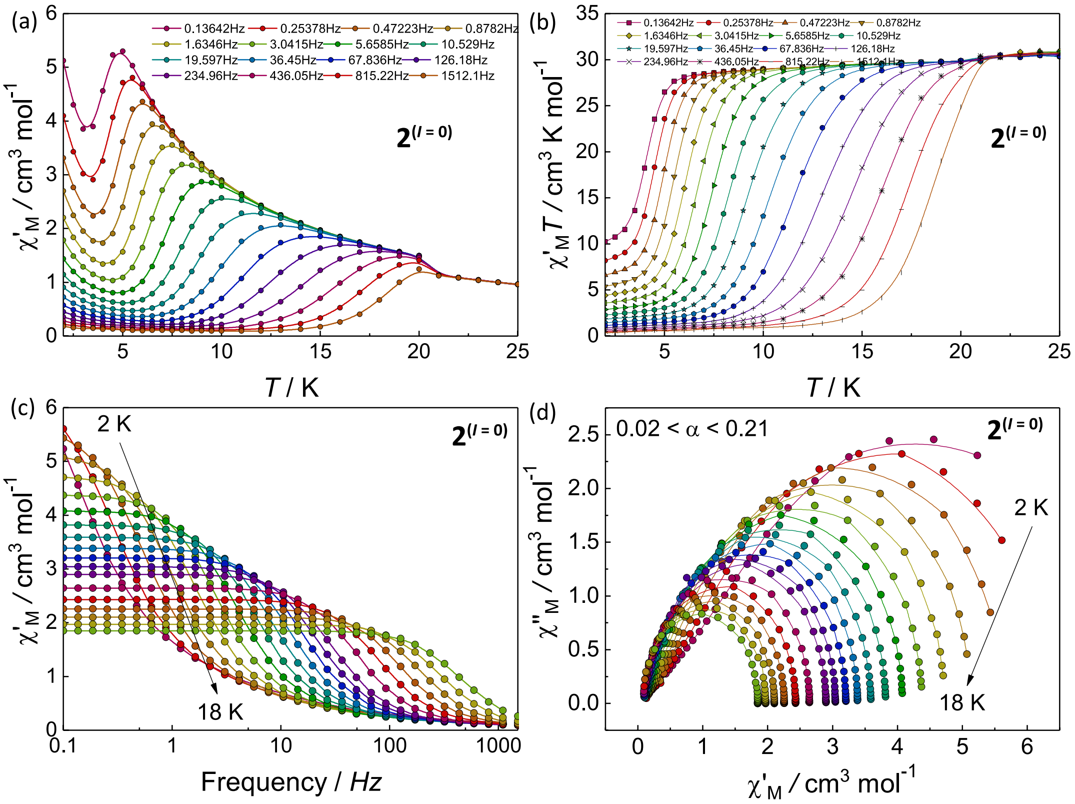

Figure S10. Dynamic magnetic data for compound $\mathbf{2}^{(I=0)}$ employing a 3.5 Oe AC field and zero DC field. (a) $\chi_M'(T)$;(b) $\chi_M''T(T)$; (c) $\chi_M'(\nu)$ and (d) Cole-Cole plots.

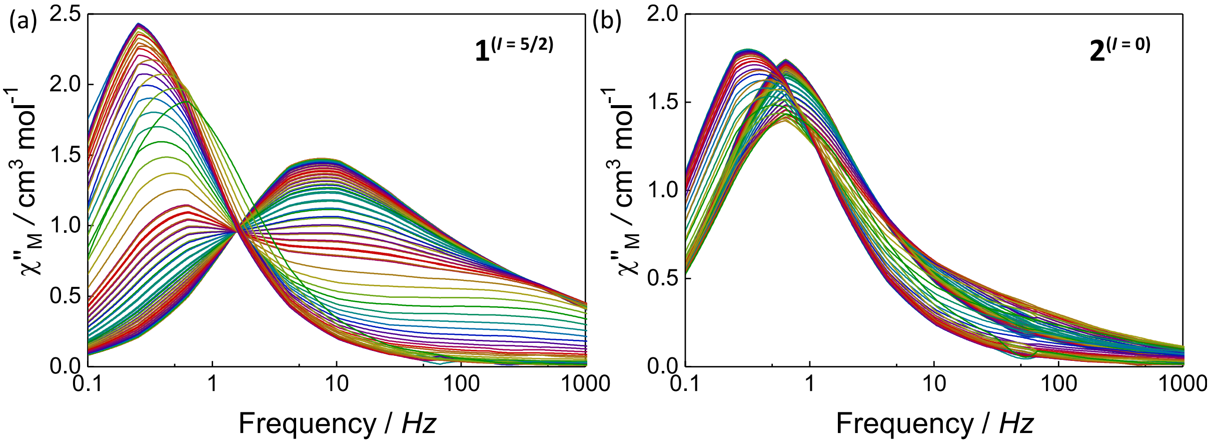

Figure S11. $\chi_M'T(\nu)$ for compounds $\mathbf{1}^{(I = 5/2)}$ (a) and $\mathbf{2}^{(I = 0)}$ (b) at different applied fields (–30 to 500 mT).
